# Design, Synthesis, and Biological Evaluation of Axitinib Derivatives

**DOI:** 10.3390/molecules23040747

**Published:** 2018-03-23

**Authors:** Na Wei, Jianqing Liang, Shengming Peng, Qiang Sun, Qiuyun Dai, Mingxin Dong

**Affiliations:** 1 Department of Chemistry, Xiangtan University, Xiangtan 411105, China; shining_xtu@163.com; 2 Lab of Protein Engineering, Beijing Institute of Biotechnology, Beijing 100071, China; theresermm@163.com

**Keywords:** axitinib, synthesis, VEGFR-2, inhibitor, tumor

## Abstract

Axitinib is an approved kinase inhibitor for the therapy of advanced metastatic renal cell carcinoma (RCC). It prevents angiogenesis, cellular adhesion, and induces apoptosis of cancer cells. Here, nine axitinib derivatives were designed by replacing the C=C moiety with the N=N group, and the substituted benzene or pyrrole analogs were considered to replace the pyridine ring. Biological activity results showed that most of nascent derivatives exhibited favorable VEGFR-2 kinase inhibitory activities, and **TM6**, **7**, **9**, and **11** behaved more potent anti-proliferative activities than axitinib. This novel series of compounds shows a potential for the treatment of solid tumors and other diseases where angiogenesis plays an important role.

## 1. Introduction

Angiogenesis is a process of new blood vessels sprouting from existing vasculature, which plays a major role in wound healing, tissue repair, and some pathological disorders like tumor growth. In cancer, angiogenesis is essential for supplying growing tumors with oxygen, nutrients, and growth factors, removing the waste products of metabolism [1]. Tumors that lack an adequate vasculature become necrotic or apoptotic and do not grow beyond a limited size. A key pro-angiogenic cytokine released by many tumor types is vascular endothelial growth factor (VEGF). The angiogenic activity of the VEGF family of proteins is mediated by two high affinity receptors, VEGFR-1 and VEGFR-2 located on vascular endothelial cells, with a third, VEGFR-3 which mediates lymphangiogenesis, found on lymphatic vessels, but VEGFR-2 is the predominant VEGF isoform responsible for the majority of downstream effects. Thus, inhibition of the VEGF signaling pathway has shown one of the most promising approaches for cancer therapy [2,3].

Axitinib (AG-013736) is a novel, potent, multi-target tyrosine kinase inhibitor that inhibits VEGFR-1, VEGFR-2, VEGFR-3, PDGFR β, and c-Kit at sub-nanomolar or nanomolar concentrations [4]. Axitinib inhibits angiogenesis, vascular permeability, and blood flow. In nonclinical models, axitinib inhibits the growth of primary tumors in human xenografts by interacting with tumor angiogenesis in colorectal, breast, melanoma, and glial tumors. It also decreased metastasis to lung and lymph nodes in murine lung and melanoma models [5]. Besides, comparing with other multi-target tyrosine kinase inhibitors (TKIs) such as sunitinib and pazopanib, axitinib is a more selective inhibitor of VEGFR and exhibit little off VEGFR-targeted effect [6,7]. Axitinib was approved by the US Food and Drug Administration (FDA) in 2012 for the therapy of advanced metastatic renal cell carcinoma (RCC).

**Figure 1:**
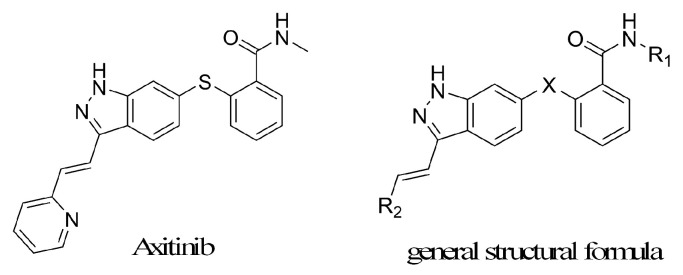
The structure of axitinib and derivatives general structural formula.

There were few public reports on axitinib derivatives, except that 7 analogs were listed in 1 paper and 30 analogs were enumerated in 1 patent [8,9]. The structure of axitinib and the known derivatives general structural formula was shown in Figure 1. In this study, a novel series of axitinib derivatives were prepared and investigated against VEGFR-2 kinase inhibitory activities and anti-proliferative activities in vitro. The structure–activity relationship of these axitinib derivatives was also discussed.

## 2. Results and Discussion

### 2.1. Chemistry

The aim of this work was to design and synthesize axitinib derivatives. The molecule design principles mainly considered convenient synthesis, improving water solubility and changing the stilbene fragment. A novel series of axitinib derivatives were designed by replacing the C=C moiety with the N=N group, and the substituted benzene or pyrrole analogs were considered to replace the pyridine ring.

There are a lot of synthetic methods to make aromatic azo compounds, but for asymmetric aromatic azo compounds, diazo-reaction, and nitroso-amino coupling reaction are the main choices [10,11,12]. Nitroso compounds are usually unstable because of easily oxidized, and it is also difficult to obtain through oxidizing amino compounds or reducing nitro-compounds. The diazo-reaction has the advantages of reacting rapidly, high yield, wide application range, easy operating and so on, so diazo-reaction was adopted to build target compounds [10,11].

**Scheme 1:**
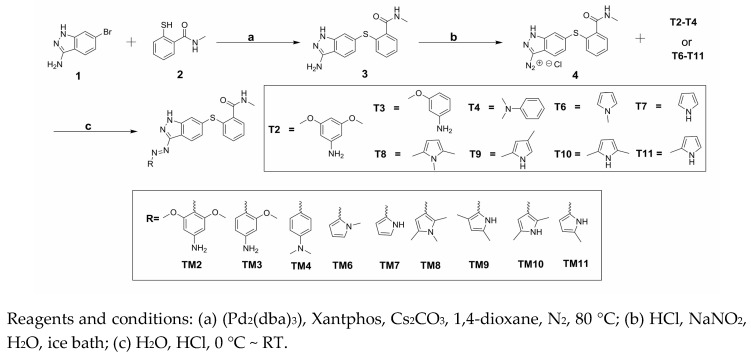
Synthesis of target compounds (**TM2**–**TM11**).

The synthesis of target compounds (**TM2**–**TM4, TM6**–**TM11**) was carried out according to the synthetic route shown in Scheme 1. High yield of **3** was achieved via Migita coupling by stirring **1** and **2** at 80 °C for 12 h [13]. **3** was diazotized, resulting diazonium salt **4** solution. Target compounds were obtained through the coupling reaction of **4** with the corresponding materials (**T2**–**T4**, **T6**–**T11**) [10]. The HR-MS spectrum and NMR spectra of the target products were described in the experimental section.

### 2.2. Biological Evaluation

A major mediator of VEGF-driven responses in endothelial cells is VEGFR-2, which is considered to play a decisive role in both physiologic and pathologic angiogenesis [14,15]. The homogeneous time-resolved fluorescence (HTRF) method was used in the assay that determined the inhibitory activities of target compounds against VEGFR-2 kinase [16,17]. Owing to VEGFR-2 overexpression in human umbilical vein endothelial cells (HUVEC) [18,19], these target compounds were further evaluated in vitro anti-proliferation activity against HUVEC. MTT assay was used in the cell assay [20,21,22]. In theory, axitinib and our target compounds have *cis*/*trans* isomer. In order to ensure the repeatability of the activity test, we choose to complete biological evaluation under the dark condition.

#### 2.2.1. VEGFR-2 Kinase Assay

**Figure 2:**
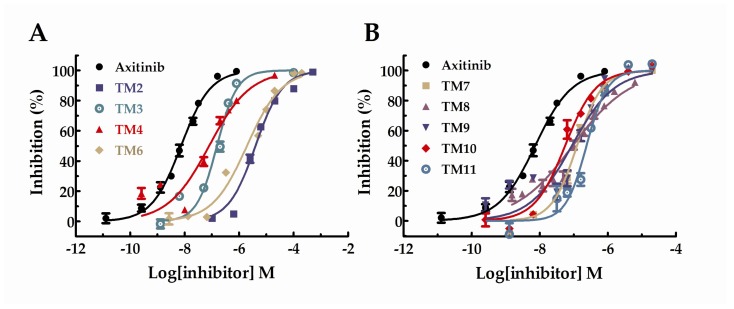
Dose–response curves for the inhibition of VEGFR-2 in the presence of axitinib derivatives.

The data was calculated and presented as IC_50_ values in Table 1. Besides, the IC_50_ curves for the compounds were shown in Figure 2. Unfortunately, the results showed that the inhibitory activities of all target compounds against VEGFR-2 were decreased to some extent compared with axitinib. Among these derivatives, **TM10** showed the highest activity with the IC_50_ value of 44 nM and **TM2** with the lowest activity (IC_50_ = 3 µM). Besides, the activity of **TM6** with the IC_50_ value of 2.29 µM was similar to **TM2**. The IC_50_ values of **TM3**, **4**, **7**, **8**, **9**, and **11** were probably between 100~330 nM, which exhibited favorable VEGFR-2 kinase inhibitory activities. All in all, most of the target compounds under investigation exhibited significant VEGFR-2 kinase inhibitory activities.

#### 2.2.2. Molecular Docking Study

**Figure 3:**
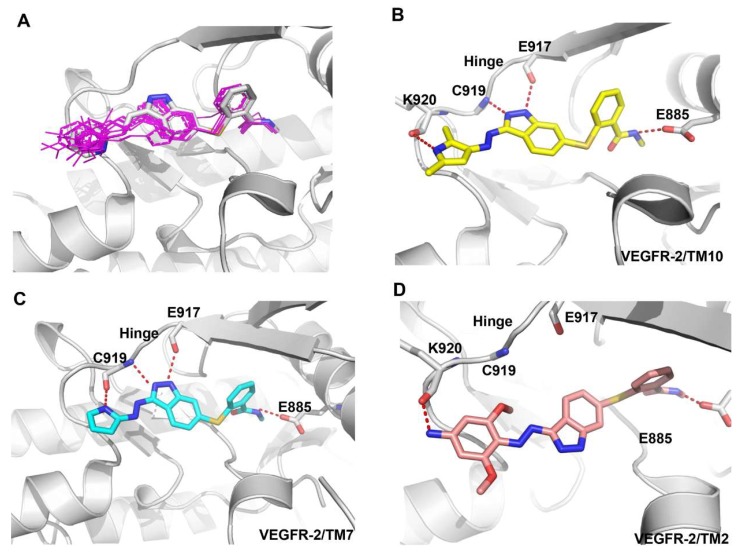
Predicted binding mode of axitinib derivatives in the VEGFR-2 active site (PDB ID Code: 3AGC). VEGFR-2 is shown in cartoon form (gray). Axitinib is depicted by sticks (carbon atoms: grey). **TM10**, **TM7**, and **TM2** are also depicted by sticks (carbon atoms: yellow, grey, and pink respectively).

To explore the structure–activity relationship, the binding modes of axitinib derivatives were predicted by using the VEGFR-2 axitinib complex (PDB ID code 4AGC) [23,24]. Compared to axitinib, axitinib derivatives filled the same binding space and adopted a similar binding conformation as shown in Figure 3A. Among the nine axitinib derivatives, **TM10** has the best kinase activity. Besides three conserved hydrogen bonds formed in VEGFR-2-axitinib complex, **TM10 f**ormed another hydrogen bond between the N-H of pyrrole group and the carbonyl oxygen of the Lys920 backbone which was important to maintain the activity (Figure 3B). Correspondingly, introduction of N-methyl group in **TM8** disturbed the formation of hydrogen bond and led to the loss of activity.

The similar observations were existed in **TM6**, **TM7**, **TM9**, and **TM11**. The N-H of pyrrole group of **TM7** also **f**ormed another hydrogen bond with the carbonyl oxygen of the Cys919 backbone which contribute to the activity (Figure 3C). In **TM9** and **TM11**, the hydrogen bond was conserved but the introduction of methyl group might be unfit for the binding cavity which caused slight loss of activity. However, **TM6** with N-methyl pyrrole group almost lost its activity because of the missing hydrogen bond.

Unfortunately, the activity of compound **TM2** was lowest, and the predicted binding mode showed that the conformation of **TM2** was different from axitinib and other axitinib derivatives. The indazole ring of **TM2** flipped to the other side due to the large volume of 3,5-dimethoxyaniline ring and lost two key hydrogen bonds which might be responsible for the drop-off loss in binding affinity (Figure 3D). Compared with **TM2**, **TM3** lacked a methoxy group but the activity had been increased by more than ten times. **TM4** lacked two methoxy groups and the activity was the best among **TM2** and **TM3**.

#### 2.2.3. HUVEC Cell Anti-Proliferation Assay

**Figure 4:**
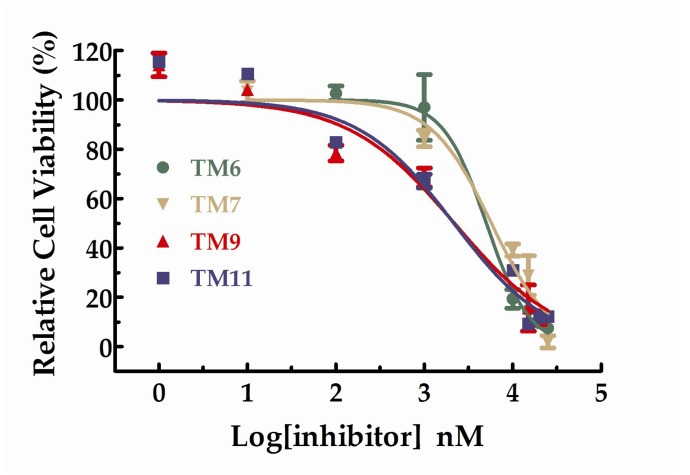
Dose–response curves for the relative cell viability of HUVEC in the presence of **TM6**,**7**,**9** and **11**.

All the compounds were tested the inhibition rations at two concentrations (1 µM and 10 µM). The experimental results were summarized in the table below. As shown in Table 2, **TM6**, **7**, **9**, and **11** exhibited inhibition ratios exceeding 60% at the concentration of 10 µM, which markedly better than axitinib. In particular, the inhibitory rate of **TM6** was nearly double that of axitinib at the concentration of 10 µM. At the concentration of 10 µM, the inhibition rations of **TM2**, **3**, and **4** did not exceed 20% and the inhibition rations of **TM8** and **9** were approaching axitinib. From Table 2, we can see that **TM6**, **7**, **9**, and **11** exhibited more potent inhibitory activities favorable anti-proliferative activities than axitinib at the concentration of 10 µM. We further studied the dose–response relationship of **TM6**, **7**, **9**, and **11** to obtain IC_50_ curves, respectively. The IC_50_ curves for the compounds were shown in Figure 4. The IC_50_ values for **TM6**, **7**, **9**, and **11** were 5.03 µM (SD = 1.13, *n* = 3), 5.73 µM (SD = 1.30, *n* = 3), 2.18 µM (SD = 1.43, *n* = 3) and 2.15 µM (SD = 1.46, *n* = 3), respectively.

It can be seen from the VEGFR-2 kinase activity and cell viability activity of target compounds that the cell activity was not consistent with the VEGFR-2 enzyme activity. The reason for this phenomenon is likely that axitinib is a multi-target tyrosine kinase inhibitor, and axitinib derivatives also exhibit potent inhibitory activities against other tyrosine kinase. Target compounds’ kinase activities against other tyrosine kinase and other cancer cell lines would be tested later.

## 3. Materials and Methods

### 3.1. Chemistry

All reagents and solvents were reagent grade or were purified by standard methods before use. The structures of these compounds were characterized by NMR and HR-MS. ^1^H-NMR spectra were recorded at 400 MHz. The structural information for both of the intermediates and target compounds is shown below. ^1^H-NMR spectra were determined on JEOL JNM-ECA400 PFT-NMR spectrometer. Chemical shifts for ^1^H-NMR are reported in parts per million (ppm) and calibrated to the solvent peak set. HR-MS were determined by an Agilent 1260 Liquid chromatography mass spectrometry spectrometer. All reactions requiring anhydrous conditions were performed under a positive nitrogen flow, and all glassware was oven dried. Isolation and purification of the compounds were performed by flash column chromatography on silica gel 60 (230–400 mesh). Analytical thin-layer chromatography (TLC) was conducted on TLC plates (silica gel 60 F254, aluminium foil) and spots were visualized by UV light and⁄or by means of dyeing reagents.

General synthetic routes of the nine target compounds were provided Scheme 1.

*2-(3-amino-1H-indazol-6-ylthio)-N-methylbenzamide* (**3**) To a nitrogen-purged reactor vessel at ambient conditions was charged 1, 4-dioxane (12 mL) followed by 6-bromo-1H-indazol-3-amine (106 mg, 0.5 mmol, 1.0 equiv), Xantphos (28.9 mg, 0.05 mmol, 0.1 equiv), tris(dibenzylideneacetone)dipalladium [Pd_2_(dba)_3_] (22.9 mg, 0.025 mmol, 0.05 equiv), and Cesium carbonate (325.8 mg, 1.0 mmol, 2.0 equiv). The reactor was purged, and the mixture was held at a target of 25 °C and stirred for about 30 min. A solution of 2-mercapto-N-methylbenzamide (87.8 mg, 0.525 mmol, 1.05 equiv) in 1,4-dioxane (8 mL) was added over about 30 min. The batch mixture was heated to a target of 80 °C. The reaction was held for 12 h. Once TLC indicated the reaction was complete, the reaction mixture was evaporated at 40 °C, dissolved in chloroform and purified on a silica column with CH_2_Cl_2_/MeOH (50:1). Obtained as a light-yellow powder 80 mg (yield: 57%). ^1^H-NMR (400 MHz, DMSO-*d*_6_) δ 11.47 (s, 1H), 8.35–8.32 (m, 1H), 7.68–7.66 (m, 1H), 7.43–7.40 (m, 1H), 7.26–7.25 (m, 1H), 7.24–7.21 (m, 1H), 7.20–7.16 (m, 1H), 6.91–6.88 (m, 1H), 6.86–6.84 (m, 1H), 5.41 (s, 2H), 2.73 (d, *J* = 4.64 Hz, 3H). ^13^C-NMR (100 MHz, DMSO-*d*_6_) δ 168.4, 149.8, 142.3, 137.1, 136.8, 131.9, 130.7, 129.8, 128.2, 126.2, 122.8, 122.0, 114.8, 114.6, 26.6. HR-MS (ESI) *m*/*z* calculated for C_15_H_14_N_4_OS [M + H]^+^ 299.0967, found 299.0963.

#### General Procedure 1

12 M HCl (0.034 mL, 0.4 mmol, 2.0 equiv) was added to a suspension of **3** (59.6 mg, 0.2 mmol, 1.0 equiv) in water (10 mL) at 0 ^o^C and the mixture was stirred for 5 min. NaNO_2_ (14.48 mg, 0.21 mmol, 1.05 equiv) in water (3 mL) was added dropwise and the resulting solution was stirred at 0 °C for 30 min, obtained diazonium salt solution. 12 M HCl (0.017 mL, 0.2 mmol, 1.0 equiv) was added to a suspension of the **T2**-**T4** or **T6-T11** (0.2 mmol, 1.0 equiv) in water (10 mL) at 0 °C, diazonium salt solution was added dropwise and the resulting solution was stirred at 0 °C for 30 min. Then saturated aqueous sodium bicarbonate solution was added dropwise till the pH was 7~8. The reaction was stirred overnight. The resulting residue was extracted with CH_2_Cl_2_ (3 × 10 mL), washed with brine, dried over Na_2_SO_4_, and concentrated under reduced pressure to afford the crude product. The crude product was purified on a silica column.

*2-(3-((4-amino-2,6-dimethoxyphenyl)diazenyl)-1H-indazol-6-ylthio)-N-methylbenzamide* (**TM2**). The synthesis was performed according to General Procedure 1. 3, 5-dimethoxyaniline (**T2**) (30.6 mg, 0.2 mmol, 1.0 equiv). The crude product was purified on a silica column with CH_2_Cl_2_/MeOH (20:1). Obtained as a red powder. 27.8 mg (yield: 30%). ^1^H-NMR (400 MHz, DMSO-*d*_6_) δ 13.15 (s, 1H), 8.38 (d, *J* = 8.4 Hz, 1H), 8.34–8.31(m, 1H), 7.49–7.48 (m, 1H), 7.45–7.43 (m, 1H), 7.29–7.20 (m, 2H), 7.15–7.12 (m, 1H), 6.99–6.97 (m, 1H), 6.20 (s, 2H), 5.96 (s, 2H), 3.77 (s, 6H), 2.74 (d, *J* = 4.6Hz, 3H). ^13^C-NMR (100 MHz, DMSO-*d*_6_) δ 168.4, 158.2 (2C), 157.4, 154.6, 142.2, 137.3, 136.4, 133.2, 130.8, 130.4, 128.3, 127.3, 126.6, 125.7, 123.1, 114.7, 113.3, 90.4 (2C), 56.2 (2C), 26.6. HR-MS (ESI) *m*/*z* calculated for C_23_H_22_N_6_O_3_S [M + H]^+^ 463.1552, found 463.1548.

*2-(3-((4-amino-2-methoxyphenyl)diazenyl)-1H-indazol-6-ylthio)-N-methylbenzamide* (**TM3**). The synthesis was performed according to General Procedure 1. 3-methoxyaniline (**T3**) (24.62 mg, 0.2 mmol, 1.0 equiv). The crude product was purified on a silica column with CH_2_Cl_2_/MeOH (20:1). Obtained as a red powder. 27.7 mg (yield: 32%). ^1^H-NMR (400 MHz, DMSO-*d*_6_) δ 13.35 (s, 1H), 8.38–8.35 (m, 1H), 8.32 (d, *J* = 8.44 Hz, 1H), 7.59 (d, *J* = 8.8 Hz, 1H), 7.53 (s, 1H), 7.47–7.45 (m, 1H), 7.30–7.22 (m, 2H), 7.18–7.16 (m, 1H), 7.03–7.01 (m, 1H), 6.32 (d, *J* = 2 Hz, 1H), 6.25–6.23 (m, 3H), 3.88 (s, 3H), 2.74 (d, *J* = 4.6 Hz, 3H). ^13^C-NMR (100 MHz, DMSO-*d*_6_) δ 168.4, 160.2, 157.0, 155.5, 142.3, 137.6, 136.0, 133.9, 133.3, 130.9, 130.7, 129.3, 128.3, 127.4, 126.8, 125.1, 117.4, 114.6, 113.4, 107.4, 96.6, 56.2, 26.6. HR-MS (ESI) *m*/*z* calculated for C_22_H_20_N_6_O_2_S [M + H]^+^ 433.1447, found 433.1442.

*2-(3-((4-(dimethylamino)phenyl)diazenyl)-1H-indazol-6-ylthio)-N-methylbenzamide* (**TM4**). The synthesis was performed according to General Procedure 1. N, N-dimethylaniline (**T4**) (24.2 mg, 0.2 mmol, 1.0 equiv). The crude product was purified on a silica column with CH_2_Cl_2_/MeOH (20:1). Obtained as a red powder. 24.1 mg (yield: 28%). ^1^H-NMR (400 MHz, DMSO-*d*_6_) δ 13.55 (s, 1H), 8.40–8.36 (m, 1H), 8.31 (d, *J* = 8.44 Hz, 1H), 7.82 (d, *J* = 9.12 Hz, 2H), 7.54 (m, 1H), 7.47–7.45 (m, 1H), 7.30–7.23 (m, 2H), 7.20–7.18 (m, 1H), 7.03–7.01 (m, 1H), 6.81 (d, *J* = 9.36 Hz, 2H); 3.02 (s, 6H), 2.74 (d, *J* = 4.64 Hz, 3H).^13^C-NMR (100 MHz, DMSO-*d*_6_) δ 168.4, 156.0, 153.0, 143.6, 142.4, 137.6, 136.8, 134.1, 130.9, 130.7, 129.3, 128.4, 127.7, 126.8, 124.9, 124.7, 114.7, 114.4, 113.2, 112.2, 40.4 (2C), 26.6. HR-MS (ESI) *m*/*z* calculated for C_23_H_22_N_6_OS [M + H]^+^ 431.1654, found 431.1650.

*N-methyl-2-(3-((1-methyl-1H-pyrrol-2-yl)diazenyl)-1H-indazol-6-ylthio)benzamide* (**TM6**). The synthesis was performed according to General Procedure 1. 1-methyl-1H-pyrrole (**T6**) (16.22 mg, 0.2 mmol, 1.0 equiv). The crude product was purified on a silica column with CH_2_Cl_2_/MeOH (20:1). Obtained as a yellow powder. 27.3 mg (yield: 35%). ^1^H-NMR (400 MHz, DMSO-*d*_6_) δ 13.5 (s, 1H), 8.35–8.32 (m, 1H), 8.21 (d, *J* = 8.4 Hz, 1H), 7.53 (s, 1H), 7.47–7.44 (m, 1H), 7.32–7.23 (m, 3H), 7.20–7.17 (m, 1H), 7.06–7.04 (m, 1H), 6.63-6.62 (m, 1H), 6.29–6.27 (m, 1H), 3.94 (s, 3H), 2.73 (d, *J* = 4.6 Hz, 3H). ^13^C-NMR (100 MHz, DMSO-*d*_6_) δ 168.4, 156.9, 147.2, 142.5, 138.0, 135.5, 134.4, 131.1, 130.9, 128.9, 128.3, 127.6, 127.0, 124.3, 114.6, 113.0, 110.8, 99.8, 33.8, 26.6. HR-MS (ESI) *m*/*z* calculated for C_20_H_18_N_6_OS [M − H]^−^ 389.1185, found 389.1187.

*2-(3-((1H-pyrrol-2-yl)diazenyl)-1H-indazol-6-ylthio)-N-methylbenzamide* (**TM7**). The synthesis was performed according to General Procedure 1. Pyrrole (**T7**) (13.41 mg, 0.2 mmol, 1.0 equiv). The crude product was purified on a silica column with CH_2_Cl_2_/MeOH (20:1). Obtained as an orange powder. 15.8 mg (yield: 21%). ^1^H-NMR (400 MHz, DMSO-*d*_6_) δ 13.47 (s, 1H), 12.03 (s, 1H), 8.38–8.35 (m, 1H), 8.26 (d, *J* = 8.4Hz, 1H), 7.51 (s, 1H), 7.47–7.45 (m, 1H), 7.31–7.23 (m, 2H), 7.20–7.17 (m, 1H), 7.08–7.07 (m, 1H), 7.04–7.02 (m, 1H), 6.92–6.91 (m, 1H), 6.33–6.31 (m, 1H), 2.73 (d, *J* = 4.6 Hz, 3H). ^13^C-NMR (100 MHz, DMSO-*d*_6_) δ 168.4, 156.1, 146.7, 142.4, 137.7, 135.7, 134.2, 130.9, 130.8, 128.4, 127.4, 126.9, 124.5, 114.7, 113.2, 111.9, 26.6. HR-MS (ESI) *m*/*z* calculated for C_19_H_16_N_6_OS [M + H]^+^ 377.1185, found 377.1179.

*N-methyl-2-(3-((1,2,5-trimethyl-1H-pyrrol-3-yl)diazenyl)-1H-indazol-6-ylthio)benzamide* (**TM8**). The synthesis was performed according to General Procedure 1. 1, 2, 5-trimethylpyrrole (**T8**) (21.82 mg, 0.2 mmol, 1.0 equiv). The crude product was purified on a silica column with CH_2_Cl_2_/MeOH (20:1). Obtained as a light orange powder. 24.3 mg (yield: 29%). ^1^H-NMR (400 MHz, DMSO-d6) δ 13.27 (s, 1H), 8.38–8.35 (m, 1H), 8.22 (d, *J* = 8.44 Hz, 1H), 7.51 (s, 1H), 7.46–7.44 (m, 1H), 7.30–7.21 (m, 2H), 7.17–7.14 (m, 1H), 7.01–6.99 (m, 1H), 6.13 (d, *J* = 1 Hz, 1H), 3.44 (s, 3H), 2.73 (d, *J* = 4.6 Hz, 3H), 2.53 (s, 3H), 2.18 (s, 3H). ^13^C-NMR (100 MHz, DMSO-*d*_6_) δ 168.29, 156.53, 142.33, 140.65, 137.57, 136.43, 135.85, 133.62, 131.45, 130.76, 130.64, 128.23, 127.09, 126.69, 124.48, 114.61, 113.23, 94.99, 30.71, 26.53, 12.59, 10.22. HR-MS (ESI) *m*/*z* calculated for C_22_H_22_N_6_OS [M + H]^+^ 419.1654, found 419.1649.

*2-(3-((3,5-dimethyl-1H-pyrrol-2-yl)diazenyl)-1H-indazol-6-ylthio)-N-methylbenzamide* (**TM9**). The synthesis was performed according to General Procedure 1. 2, 4-dimethyl-1H-pyrrole (**T9**) (19.02 mg, 0.2 mmol, 1.0 equiv). The crude product was purified on a silica column with CH_2_Cl_2_/MeOH (20:1). Obtained as an orange powder. 15.4 mg (yield: 19%). ^1^H-NMR (400 MHz, DMSO-*d*_6_) δ 13.22 (s, 1H), 11.54 (s, 1H), 8.38–8.35 (m, 1H), 8.26 (d, *J* = 8.48 Hz, 1H), 7.50 (s, 1H), 7.46–7.44 (m, 1H), 7.30–7.22 (m, 2H), 7.16–7.14 (m, 1H), 7.03–7.00 (m, 1H), 5.93 (d, *J* = 1.36 Hz, 1H), 2.73 (d, *J* = 4.64 Hz, 3H), 2.32 (s, 3H), 2.20 (s, 3H). ^13^C-NMR (100 MHz, DMSO-*d*_6_) δ 168.4, 156.6, 142.4, 141.5, 137.6, 136.0, 135.1, 133.6, 130.9, 130.7, 129.5, 128.3, 127.2, 126.8, 124.7, 114.7, 113.4, 95.0, 26.6, 13.4, 11.0. HR-MS (ESI) *m*/*z* calculated for C_21_H_20_N_6_OS [M − H]^−^ 403.1341, found 403.1344.

*2-(3-((2,5-dimethyl-1H-pyrrol-3-yl)diazenyl)-1H-indazol-6-ylthio)-N-methylbenzamide* (**TM10**). The synthesis was performed according to General Procedure 1. 2, 5-dimethyl-1H-pyrrole (**T10**) (19.02 mg, 0.2 mmol, 1.0 equiv). The crude product was purified on a silica column with CH_2_Cl_2_/MeOH (20:1). Obtained as a yellow powder. 29.9 mg (yield: 37%). ^1^H-NMR (400 MHz, DMSO-*d*_6_) δ 13.25 (s, 1H), 11.16 (s, 1H), 8.38–8.34 (m, 1H), 8.22 (d, *J* = 8.44 Hz, 1H), 7.51 (s, 1H), 7.46–7.44 (m, 1H), 7.30–7.21 (m, 2H), 7.16–7.14 (m, 1H), 7.01–6.99 (m, 1H), 6.03 (m, 1H), 2.73 (d, *J* = 4.56 Hz, 3H), 2.49 (s, 3H), 2.15 (s, 3H). ^13^C-NMR (100 MHz, DMSO-*d*_6_) δ 168.4, 156.8, 142.8, 142.4, 137.6, 136.5, 136.0, 133.8, 130.9, 130.7, 128.3, 127.0, 126.8, 124.8, 114.7, 113.5, 112.1, 66.89, 26.6, 13.6, 11.4. HR-MS (ESI) *m*/*z* calculated for C_21_H_20_N_6_OS [M + H]^+^ 405.1498, found 405.1494.

*N-methyl-2-(3-((5-methyl-1H-pyrrol-2-yl)diazenyl)-1H-indazol-6-ylthio)benzamide* (**TM11**). The synthesis was performed according to General Procedure 1. 2-methyl-1H-pyrrole (**T11**) (16.22 mg, 0.2 mmol, 1.0 equiv). The crude product was purified on a silica column with CH_2_Cl_2_/MeOH (20:1). Obtained as a light-yellow powder. 11.7 mg (yield: 15%). ^1^H-NMR (400 MHz, DMSO-*d*_6_) δ 13.33 (s, 1H), 11.84 (s, 1H), 8.37 (d, *J* = 4.48 Hz, 1H), 8.24 (d, *J* = 8.52 Hz, 1H), 7.50 (s, 1H), 7.46–7.44 (m, 1H), 7.30–7.22 (m, 2H), 7.14 (d, *J* = 12 Hz, 1H), 7.01 (d, *J* = 7.64 Hz, 1H), 6.83 (s, 1H), 6.08 (s, 1H), 2.73 (d, *J* = 4.48 Hz, 3H), 2.25 (s, 3H). ^13^C-NMR (100 MHz, DMSO-*d*_6_) δ 168.4, 156.3, 145.8, 142.4, 137.6, 136.2, 135.9, 133.9, 130.9, 130.7, 128.3, 127.1, 126.8, 124.7, 114.7, 113.4, 111.2, 26.6, 13.6. HR-MS (ESI) *m*/*z* calculated for C_20_H_18_N_6_OS [M − H]^−^ 389.1185, found 389.1190.

### 3.2. VEGFR2 Kinase Assay

Axitinib was purchased from TargetMol (Beijing, China). VEGFR-2 (KDR) was purchased from Carna Biosciences (Minatojima-Minamimachi, Japan). Cisbio’s HTRF KinEASE-TK Kit was used to test the enzyme inhibitory activity. Axitinib was used as a control. This method makes use of a unique substrate that contains a single phosphorylation site identified by a europium cryptate (Eu(K))-labeled antibody to phosphotyrosine [25]. Experimental operation was according to the instruction of the kit. The HTRF KinEASE-TK assay format involves two steps: the enzymatic step and the detection step. In the enzymatic step, 4 µL of compound (or kinase buffer), 2 µL (0.0123 ng/µL) of VEGFR-2 kinase solution, 2 µL (1 µM) of TK biotin substrate, and 2 µL of ATP (3 µM) were added to each well for incubation at room temperature (22–26 °C) to start the reaction, which was run for 40 min. In the detection step, detection reagents including 5 µL of streptavidin-XL665 (SA-XL665) (0.125 µM) in EDTA and 5 µL TK Antibody-Eu(K) (0.125 µM) in EDTA were added to each well and incubated for 12 h at room temperature (22–26 °C). The Molecular Devices spectra Max i3 platform HTRF detection module was used to detect the signal. The fluorescence is measured at 620 nm (Cryptate) and 665 nm (XL665). A ratio was calculated (665/620) for each well. Target compounds were dissolved into different concentrations with kinase buffer from 5 mM stock solutions in DMSO, respectively. The GraphPad Prism 5.0 software was used to draw nonlinear regression model and calculate IC_50_ values for each compound. Each test was repeated three times.

### 3.3. Molecular Docking Study

The VEGFR-2−axitinib protein−ligand complex crystal structure (PDB (4AGC) was chosen as the template to compare the docking mode among **TM2**-**TM4** and **TM6**-**TM11**. The molecular docking procedure was performed in accordance with the Glide protocol of Schrödinger. For ligand preparation, the 3D structures of axitinib derivatives were generated and minimized using Ligand prepare protocol. Hydrogen atoms were added for enzyme preparation, and OPLS2005 force field was employed. VEGFR-2 enzyme was defined as receptors, and the site sphere was selected on the basis of the ligand binding location of axitinib. The axitinib molecule was then removed, and axitinib derivatives were placed during the molecular docking procedure. The types of interactions between the docked enzyme and the ligands were analyzed at the end of the molecular docking.

### 3.4. HUVEC Cell Proliferation Assay

SV40T transforms human umbilical vein endothelial cells (PUMC-HUVEC-T1) were gifts from Dr. Lihua Ding (Institute of Biotechnology, Beijing, China). MTT Cell Proliferation and Cytotoxicity Assay Kit (#GK-5605, Genview) was used to measure cell viability and proliferation. Metabolically active cells in part by the action of dehydrogenase enzymes reduced the yellow tetrazolium MTT to generate purple reducing equivalents such as NADH and NADPH. The resulting intracellular purple formazan can be solubilized and quantified by spectrophotometer.

For MTT assay, cells were seeded into separate 96-well plates at 5000 cells/well and cultured in an incubator containing 5% CO_2_ at 37 °C for 24 h. Subsequently, target compounds were dissolved into different concentrations with culture medium from 10 mM stock solutions in DMSO, respectively. Then target compounds were added and incubated at 37 °C for 72 h. All assays were performed in triplicate. The untreated groups were used as a vehicle of control. Following the 72-h incubation, discarded culture medium and washed with PBS once time followed by adding 180 µL of fresh culture medium. Cell viability was quantified with MTT assay. According to the manufacturer instructions, 20 µL of MTT solution that was previously thawed and equilibrated to room temperature was added to each well and plates were incubated at 37 °C for 4 h. Termination of culture, the supernatant was carefully discarded and added 150 µL of Formazan dissolution for 10 min vibration at room temperature. The OD value of each well was measured by a spectrophotometer at 490 nm and the inhibitory rates expressed as percentages of the vehicle control (100%). Data processing: ratio = Vsample/Vcontrol × 100%. Vsample is for compound treatment group while Vcontrol is for solvent control group. The GraphPad Prism 5.0 software was used to draw nonlinear regression model and calculated IC_50_ values.

## 4. Conclusions

In summary, a series of novel target compounds were synthesized and evaluated in vitro for VEGFR-2 kinase inhibitory activity and potential anticancer activity against HUVEC. Most of the nascent compounds under investigation exhibited significant VEGFR-2 kinase inhibitory activity. Some derivatives showed excellent anti-proliferative activity against HUVEC comparing with axitinib. The findings highlighted the potential of this class of derivatives as more effective anticancer agents. Further detailed research will be conducted to evaluate the other VEGFR kinase inhibitory activity of these compounds. In addition, future work will confirm if these compounds inhibit other cells in vitro. There may be a need for further animal experiments.

## Figures and Tables

**Table 1:** Inhibitory activity of axitinib derivatives against VEGFR-2 kinase.

Compound	IC_50_ (nM) ± SD (*n* = 3)
**Axitinib**	7.3 ± 1.07
**TM2**	3000 ± 1.11
**TM3**	195 ± 1.11
**TM4**	135 ± 1.25
**TM6**	2290 ± 1.17
**TM7**	123 ± 1.08
**TM8**	158 ± 1.14
**TM9**	201 ± 1.26
**TM10**	44 ± 1.15
**TM11**	330 ± 1.14

**Table 2:** In vitro anti-proliferative activity assay results of target compounds in HUVEC.

Compound	Relative Cell Viability (%) ± SD (*n* = 3)	
	1 µM	10 µM
**Axitinib**	79.97 ± 6.64	53.22 ± 9.11
**TM2**	106.56 ± 1.29	94.40 ± 5.01
**TM3**	125.46 ± 8.92	84.14 ± 6.83
**TM4**	94.12 ± 1.89	82.76 ± 5.32
**TM6**	96.95 ± 10.44	19.33 ± 3.82
**TM7**	84.36 ± 4.35	39.13 ± 2.49
**TM8**	92.34 ± 9.05	61.26 ± 10.94
**TM9**	68.54 ± 0.29	32.20 ± 1.99
**TM10**	99.29 ± 0.78	66.22 ± 4.43
**TM11**	67.03 ± 0.19	30.97 ± 0.61

^a^ Control group was performed with corresponding amount of DMSO.
